# Using Decision Tree Aggregation with Random Forest Model to Identify Gut Microbes Associated with Colorectal Cancer

**DOI:** 10.3390/genes10020112

**Published:** 2019-02-01

**Authors:** Dongmei Ai, Hongfei Pan, Rongbao Han, Xiaoxin Li, Gang Liu, Li C. Xia

**Affiliations:** 1 Basic Experimental of Natural Science, University of Science and Technology Beijing, Beijing 100083, China; 2 School of Mathematics and Physics, University of Science and Technology Beijing, Beijing 100083, China; 18813128340@163.com (H.P.); rongbaohan@163.com (R.H.); lixiaoxinustb@sina.com (X.L.); s20180807@xs.ustb.edu.cn (G.L.); 3 Department of Medicine, Stanford University School of Medicine, 269 Campus Dr., Stanford, CA 94305, USA

**Keywords:** microbial relative abundances, random forest, colorectal cancer, microbial community analysis, mutual information

## Abstract

The imbalance of human gut microbiota has been associated with colorectal cancer. In recent years, metagenomics research has provided a large amount of scientific data enabling us to study the dedicated roles of gut microbes in the onset and progression of cancer. We removed unrelated and redundant features during feature selection by mutual information. We then trained a random forest classifier on a large metagenomics dataset of colorectal cancer patients and healthy people assembled from published reports and extracted and analysed the information from the learned decision trees. We identified key microbial species associated with colorectal cancers. These microbes included *Porphyromonas asaccharolytica*, *Peptostreptococcus stomatis*, *Fusobacterium,* *Parvimonas* sp., *Streptococcus vestibularis* and *Flavonifractor plautii.* We obtained the optimal splitting abundance thresholds for these species to distinguish between healthy and colorectal cancer samples. This extracted consensus decision tree may be applied to the diagnosis of colorectal cancers.

## 1. Introduction

Many microbial communities cohabit the human body, among which microbiota in the gut is the richest with more than 1000 species [1]. Gut microbes participate in many important physiological processes, such as food digestion, metabolism and immune response. In the long-term process of natural evolution, a dynamic balance has always been struck among gut microbiota, host and environment. Changes in the structure, composition and function of the microbiota lead to abnormal metabolites of gut microbes, causing, in turn, metabolic diseases, such as obesity [2] and diabetes [3], in addition to chronic gut infections, like inflammatory bowel disease [4], ulcerative colitis, and Crohn’s disease [5,6], and malignant digestive system tumors, such as colorectal [7,8] and gastric cancers [9].

Colorectal cancer (CRC) is the third most prevalent malignant tumor in the world. Deaths from colon and rectal cancers account for >9% of total cancer-related deaths [10]. Early detection can significantly improve the overall survival of CRC patients. Colonoscopy is by far the most accurate colon cancer diagnosis method but, because of the discomfort caused by its intrusiveness, many people are reluctant to undergo the procedure [11,12]. The fecal occult blood test (FOBT) [13] is another clinical tool for identifying colorectal cancer, which has the advantages of being both noninvasive and economical. However, since the accuracy of the FOBT test is relatively low, it has not been widely accepted.

Progress has been made in studying the relationship between microbes and cancers. Studies have reported signature microbial species as indicators for the early diagnosis of CRC [14,15,16]. In 2014, Zackular et al. leveraged a logit regression model, based on age, gender, race/ethnicity, Body Mass Index (BMI), and drug usage, and significantly improved the model’s accuracy in predicting colorectal adenoma by adding microbiota data [17]. Georg Zeller et al. used the nonparametric Wilcoxon rank-sum test to analyze the differentially abundant gut microbes of healthy people versus CRC patients [18]. Based on the obtained data, they developed an operational taxonomic units (OTUs)-based logistic regression classifier using least absolute shrinkage and selection operator (LASSO) regulation [19]. Their classifier performed more accurately than FOBT and, when combined with FOBT, it showed high specificity with increased sensitivity of 45% over FOBT alone [18].

In 2015, Feng et al. compared the fecal samples of cancer patients and healthy people. They used the Kruskal-Wallis test to identify differentially abundant metagenomic genes, clustered those genes into metagenomic linkage groups (MLGs), and constructed a random forest classifier with MLGs as the features. In doing so, they established the feasibility of diagnosing CRC by using only fecal microbial structures [20]. In addition to the above classifiers, support vector machines (SVMs) [21], Bayesian networks [22] and other models have been used for this task. Such research has increased awareness of the potential for screening for CRC using gut microbiota. This awareness has, in turn, spurred the ongoing search for more predictive diagnostic models [21].

Most existing models perform classification based on OTU and MLG data and have shown some success in diagnosing cancer. Of the existing classification models, the random forest classifier typically had a superior area under the curve (AUC) score. It also showed better generalizability and robustness [23], making it suitable for use with high-dimensional data. In this paper, mutual information [24,25] is introduced to remove species that are weakly associated with CRC. As a result, the quality of input data is better, thereby improving random forest classifier’s accuracy. We also extracted decision tree data from the random forest and identified the microbes that were the best predictors of the disease, thus providing a new tool for screening CRC.

Our analysis also took advantage of the available high-throughput whole genome shotgun (WGS) metagenomics data and a highly accurate relative abundance estimation algorithm-GRAMMy [26]. Accurate estimation of microbial abundance is the basis of achieving high classification accuracy. 16S rRNA-based and OTU-based microbiome analysis showed limited resolution and sensitivity [27] for abundance estimation. GRAMMy was based on a probabilistic mixture model which explicitly models the ambiguity inherent in the reference assignment of short reads, the great variation of microbial genome sizes and homologous gene copies and handles all of them well. Thus, GRAMMy can estimate relative abundance with high accuracy, enabling high-fidelity downstream analysis.

## 2. Materials and Methods

### 2.1. Mutual Information-Based Feature Selection

The number of gut microbes cells is in the same order as the number of human cells [28]. Although a large number of those microbes participate in biochemical reactions, most are not significantly different between patients and healthy people. Improper handling of those background microbes can lead to incorrect modeling, affecting efficiency and accuracy. In particular, when the random forest model is used to study the relationship between CRC and microbiota, it can lead to a high false-negative rate [17]. For small datasets, these background features can be removed intuitively and manually. With the development of metagenomics sequencing technology, the data feature space expands rapidly. Those manual removal of weakly correlated and/or irrelevant features can no longer meet the requirements of accurate classification algorithms. In this study, we proposed a mutual information criterion to filter out species with weak associations with CRC in order to improve the quality of input data and reduce the complexity of resulting classification models [29].

First, let x be a microbial species and y be the health or disease state (CRC) of a given sample. Let $P\left(x_{i}\right)$ be the probability that the microbe has an abundance level of $x_{i}$ and, likewise, let $P\left(y_{i}\right)$ be the probability that the sample is in the state of $y_{i}$. $P(x_{i}|y_{j})$ denotes the probability that microbe x has an abundance level of $x_{i}$, given that the sample is in the state of $y_{i}$. For a given microbial species, the information entropy, H(x), and the conditional entropy, H(x|y), are defined as

(1) $$H\left(x\right)=-\underset{i}{\sum }P\left(x_{i}\right)\log p\left(x_{i}\right)$$

(2) $$\mathrm{and}\;H\left(x|y\right)=-\sum_{j}P\left(y_{i}\right)\sum_{i}P\left(x_{i}|y_{j}\right)\log p\left(x_{i}|y_{j}\right),$$

assuming that the disease state of a given sample is known.

Based on H(x) and H(x|y), one can obtain the mutual information of the microbe x and the sample’s disease status y, MI(x,y), as

(3) $$\begin{array}{cc} MI(x,y) & =H(x)-H(x|y)\\ & =H(y)-H(y|x)\\ & =\sum_{x,y}p(x,y)\log\frac{p(xy)}{p(x)p(y)} \end{array}$$

Then, one can obtain a standardized similarity measure between the microbe x and the disease state y.

(4) $$Sim\left(x,y\right)=2\left[\frac{MI\left(x,y\right)}{H\left(x\right)+H\left(y\right)}\right]$$

This similarity, Sim(x,y), has a value range of [0,1]. A value of 1 indicates that the microbe is sufficiently informative for the disease status, and a value of 0 indicates that the microbe is completely independent, or uninformative, for the disease status. Commonly, similarity is a number between 0 and 1. Thus, we can remove microbes that have a weak association with the disease status by filtering out features have MI below a specified similarity threshold to ensure data compactness.

### 2.2. Workflow for Metagenomics Analysis

**Figure 1:**
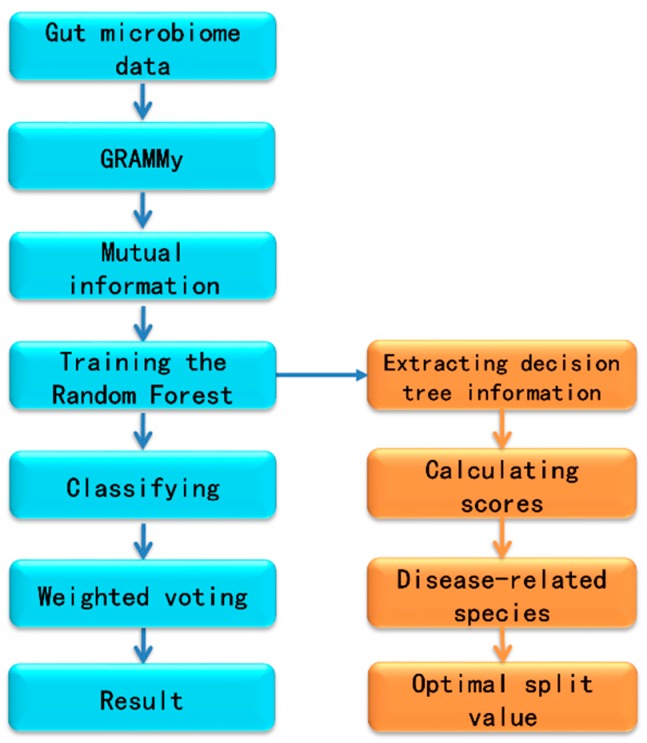
The workflow for metagenomic analysis.

The bioinformatics workflow we implemented for metagenomic analysis was shown in Figure 1. First, we downloaded the metagenomic gut microbiome data of colorectal cancer and a comprehensive set of microbial reference sequences from the NCBI. The relative abundance of gut microbial species were estimated using GRAMMy. We used mutual information to screen features and filter out weakly correlated, or unrelated, microorganisms, as well as redundant features, then trained and tested random forest models based on the filtered estimated microbial abundance data. All decision tree classification results were combined using a weighted voting method. The information of all random forest generated decision trees was extracted and analyzed. It included the frequencies of different microbes and their positions in the decision trees. We computed the voting weights as the correlation between microbes and colorectal cancer. We then aggregated the splitting values of species abundance for all decision trees and averaged them to obtain an unbiased estimate of the optimal splitting value for the differentiating threshold for each species. All data, along with the bioinformatics pipeline to reproduce the results described in this paper, can be freely accessed from GitHub at https://github.com/gutmicrobes/metaRF.git.

### 2.3. Extracting the Information of Decision Trees from Random Forest

A random forest-based disease screening model was trained to classify healthy and disease samples in the dataset. However, beyond classification, it is not previously possible to draw conclusions about which features would have greater effects on the results from the trained random forest model. This short come can be attributed to the formulation of random forest model which is typically treated as a black box [30], that is a random forest is comprised of a large number of decision trees in each of which a set of randomly selected features are used to fit the training data [31]. The introduction of stochasticity in building the random forest has, correspondingly, introduced significant complexity for understanding the decision process.

**Figure 2:**
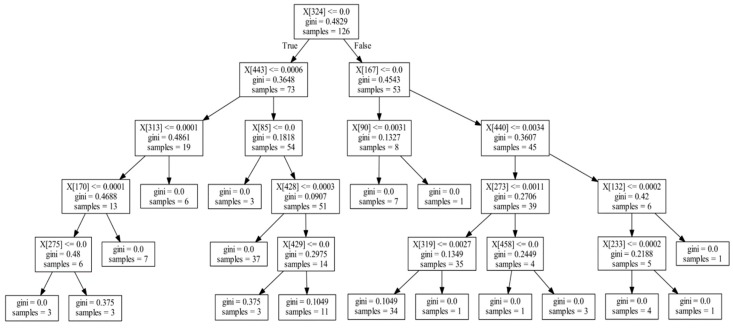
Schematic diagram of one decision tree. Decision trees have nodes and every node includes a feature ID (microbe ID), split value, Gini index, and sample number.

Although randomness is introduced in the random forest, determinism could be traced [32]. One way to achieve it is by extracting the underlying decision trees. After the random forest is trained, all node contents, partitioning method, and threshold values of individual decision trees are kept. Post-training, then, it is possible to extract and analyze these data to create a faithful description of the black box fitting process. To accomplish this, we proposed a method of extracting information from the decision trees of the random forest. More specifically, we first infer features informative to disease status by mining and analyzing all node data to identify microbes most predictive of disease status and learn their corresponding splitting threshold values. Then, when random forest training is completed and the structures of all decision trees have been determined, we perform feature extraction of these microbes. Figure 2 shows a diagram of a typical decision tree inside the trained random forest. Each box contains the feature ID (microbe ID), split threshold, sample numbers, and other information about the node.

The position of some features, e.g., the relative distance from the root, in the decision tree reflects the strength of association between microbes and diseases. For example, in Figure 2, feature X[324] is the optimal splitting feature, found via the Gini index, in the training set of the decision tree (samples = 126). Features X[443] and X[167] in the second layer are the optimal splitting features found for the subsets (samples = 73, 53) after the first splitting; although, they are weaker than the feature X[324] in the first layer. Likewise, the features found in the third layer are weaker still than those in the second layer in association. Based on these ranks, we can measure the strength of association between microbes and disease, by summarizing them for each feature over all decision trees. We termed this process the informative rank analysis within the random forest model.

To perform the informative rank analysis, we enumerated the nodes from the tree structure in Figure 2 in tabular format as Table 1. The columns: Tree_ID is the decision tree index in the random forest, Node_Index is the node index within the decision tree, Father is the parent node of the indexed node in the tree, and Layer is the layer’s depth where the node is located in the decision tree. Microbe_ID and Split_Value refer to the optimal feature ID and split value selected by computing the Gini coefficient, respectively. Sample_Number shows the number of samples for which the feature is included, which measures the importance of the feature. After aggregating the data into this tabular format, we counted the position and number of occurrences of each feature in all decision trees.

Taking feature X[324] as an example (Table 2), it has 151 Layer 0 occurrences, 156 Layer 1 occurrences, and 155 Layer 2 occurrences in the decision tree. Based strata data similar to this, we compute a score indicating the effect of feature on the disease, which is described below.

We assumed that a random forest has a total of N decision trees and that each decision tree has L layers. The sum of the node numbers in the lth layer is denoted as $N_{l}$, and the sample number at the ith node of the lth layer is denoted $a_{li}$. First, the average number of participating decision samples for the nodes in the lth layer is calculated as

(5) $$\bar{a_{l}}=\frac{1}{n_{l}}\overset{N_{l}}{\underset{i=1}{\sum }}a_{li}$$

Hence, we assigned a weight to the lth layer.

(6) $$v_{l}=\frac{\bar{a_{l}}}{\sum_{l=1}^{L}\bar{a_{l}}}$$

With the weight for lth layer in combination $c_{jl}$, which is the occurrence number of a feature $c_{j}$ at lth layer, the effect score ($c_{j\cdot score}$) of feature j is calculated as

(7) $$c_{j\cdot score}=\overset{L}{\underset{l=1}{\sum }}c_{jl}\times v_{l}$$

Finally, according to the ranking based on this overall effect score, the microbial species associated with the CRC were identified.

To understand the microbes associated with CRC, we further analyzed the abundance split threshold values for the pivotal microbes in the decision trees. Every time a microbe feature was selected by a decision tree to split the samples, the optimal abundance threshold of this microbe was also determined. This abundance threshold represented the optimal split point for abundance of the microbe between healthy samples and diseased samples, a tipping point while more or less its abundance predicts healthy samples or diseased samples. To obtain a generalizable splitting value, we counted the number of all decision trees ($N_{c_{k}}$) with features selected as the first splitting feature, and the corresponding splitting abundance threshold are. Then, the averaged splitting abundance threshold ($a_{c_{k}}$) can be derived as

(8) $$a_{c_{k}}=\frac{1}{N_{c_{k}}}\overset{N_{c_{k}}}{\underset{n=1}{\sum }}a_{nc_{k}}$$

### 2.4. Training and Testing Datasets

The data used in this paper were obtained from the NCBI (National Center for Biotechnology Information), as shown in Table 3. Dataset F [18] consists of 156 samples from France (61 adenoma samples, 42 adenoma patient samples, and 53 CRC patient samples). The adenoma samples were divided into two groups according to tumor size. The number of samples with an adenoma diameter smaller than 10 mm (small) was 27, and the number of samples with an adenoma diameter larger than 10 mm (large) was 15. The CRC samples were divided into two parts according to the American Joint Committee on Cancer (AJCC) cancer staging system (forth version) [33]: early stage (0, I, II) and late stage (III, IV). The number of samples were 22 and 31 for the two stages, respectively. The metagenomics data were obtained from the European Nucleotide Archive (ENA) and NCBI databases (accession number ERP005534). Another dataset, A, included 156 samples from Austria, including 61 healthy samples, 47 adenoma patient samples (both sizes), and 46 colorectal cancer patient samples (both stages). The metagenomics data of this dataset were downloaded from the ENA and NCBI databases (accession number ERP008729) [18].

First, relative abundance data of microbes from stool samples of healthy people and CRC patients were selected from dataset F and dataset A, respectively, with dataset F having 124 samples and dataset A having 99 samples. The small number of training samples necessitated feature selection to avoid trapping by local minimum and overfitting. The mutual information index for each feature was calculated, and the top 300 mutual information index ranked features were selected from nearly 600 features to generate the filtered input data. We used a 6-fold cross-validation method for training classification models. We did the cross-validation with data rotation using 5/6 of data for training and the remaining 1/6 data for testing in each rotate. In each round of training, 1000 decision trees were generated with a maximum allowed tree depth of 5.

## 3. Results and Discussion

### 3.1. Informative Rank Extraction from Decision Tree

The random forest classifier, which is a multi-classification integration system, is often considered a black box model, as previously noted. It concerns itself with the identification rate, but does not elect to describe the decision process [34]. However, after training a random forest, we know the every detail of the forest, such as what features were used to split each node, as well as their applicable threshold values and efficiencies. We analyzed the gut microbes and microbial abundance thresholds closely related to CRC based on these details. Table 4 lists the 14 microbes with high scores and abundance thresholds obtained according to Equation (3). These microbes were predicted as the most closely associated with CRC. Our finding was consistent with the findings of many existing studies. For example, Zeller and Feng et al. showed that *Porphyromonas asaccharolytica*, *Peptostreptococcus stomatis*, *Fusobacterium vincentii,* and *Fusobacterium animalis* were predictive in the early diagnosis of colorectal cancer, which are among the tops of our list; in particular, *F. nucleatum* is a common CRC-related species which promotes tumor development [18,20,35]. In addition to these well-known correlated species, Mancabelli L et al. showed that *Parvimonas sp., Streptococcus vestibularis* and *Flavonifractor plautii,* all of which had high scores, in our list and were associated with CRC [36]. Further experiments are required to elucidate their mechanistic implications.

In addition to identifying the microbes closely associated with CRC, we also obtained the optimal splitting relative abundance thresholds of gut microbes between both the control and disease datasets, as shown in Table 4. These abundance thresholds of special gut microbes may be important for the diagnosis and treatment of CRC. That is, knowing the abundance of a specific microbe through analysis of a sample data enables the potential for more accurate disease diagnosis. According to these abundance thresholds, we calculated the proportion of CRC patients in the sample, making it possible to extrapolate the probability of being diseased beyond these thresholds. For instance, when the relative abundance of *Porphyromonas asaccharolytica* is greater than 3.052 × 10^−5^, the percentage of CRC among all samples exceeds 88%, or when the abundance of *Peptostreptococcus stomatis* is greater than 9.154 × 10^−5^, the percentage of CRC among all samples exceeds 90%. In addition, the percentage of CRC in cases in which *Fusobacterium 7* was detected exceeds 85%. As noted, such information may allow for an earlier diagnosis of the disease and as well as more options for treating disease. Specifically, according to the abundance characteristics of gut microbes, fecal transplantation may be performed for the targeted adjustment of some microbes with abnormal abundance in order to abate the development of, or even cure, the disease in conjunction of other therapies. Further research needs to be conducted to test such hypothetical interventions in a clinical setting.

### 3.2. Top 20 Microbial Species with High Relative Abundance

**Figure 3:**
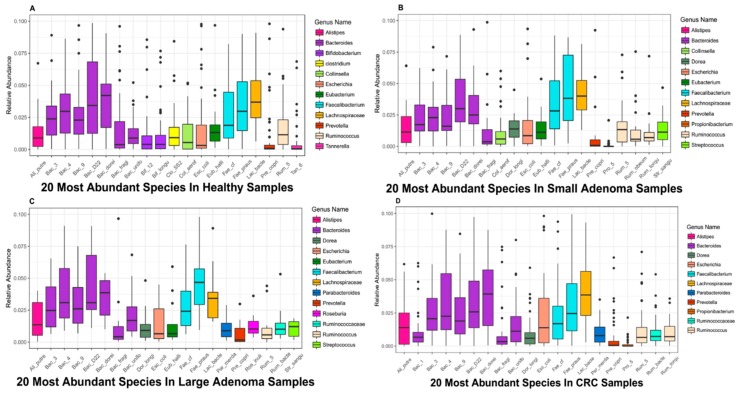
Top 20 microbial species with high relative abundance in samples of different disease stages. (**A**) The relative abundances of the top 20 microbes in the healthy samples. (**B**) The relative abundances of the top 20 microbes in small adenoma patients. (**C**) The relative abundances of the top 20 microbes in large adenoma patients. (**D**) The relative abundances of the top 20 microbes in colorectal cancer patients. The horizontal axis represents the abbreviation of the corresponding names of microbial species, the vertical axis represents the relative abundance of the corresponding microbes, and the different colored bars in the box plot indicate different microbial genera in Figure 3A–D.

To conduct a more in-depth study of the relationship between colorectal cancer patients and microbes, this section uses boxplots to show the average relative abundances of the top 20 microbes in the healthy control group, small adenoma patients, large adenoma patients, and colorectal cancer patients (Figure 3).

It can be seen from Figure 3 that, several microbial species belong to the *Bacteroides* genera ranked in the top twenty, demonstrating that *Bacteroides* play a pivotal role in the human gut. Notably, a study has shown that the *Bacteroides* genera plays an important role in the human gut digestion process and is closely related to human health [37].

By comparing the boxplots at different disease stages, we found some microbes are in abundance in healthy samples but, in patients with disease samples, the abundance of those microbes decreases. For example, multiple microbes in the genus of *Bifidobacterium* ranked in the top 20 most abundant microbes in the healthy samples but the abundance was significantlyreduced in the adenoma and cancer patients, in a way that could not be seen any more in the boxplot. *Bifidobacterium* is an important probiotic and, in conjunction with other microbes, can have a range of beneficial health effects, regulating gut microbiota balance, inhibiting pathogens and pathogenic bacteria that infect intestinal mucosa, inhibiting the activities of many carcinogenic enzymes, improving gut mucosal barriers, and reducing intestinal lipopolysaccharide levels [38]. In addition, *Bifidobacterium* has been clinically demonstrated to be used as a probiotic in the treatment of ulcerative colitis and has a beneficial effect on maintaining and alleviating the disease [39]. It has, therefore, been widely used in the food industry and pharmaceutical fields.

Similar to *Bifidobacterium*, Clostridium is only present in Figure 3A, yet not in the other three figures. It also has a very important impact on human health. A study of Brüggemann has shown that some non-pathological *Clostridium* strains may help treat diseases such as cancer; it can selectively target cancer cells, invade solid tumors and self-replicate. Therefore, *Clostridium* can be used to deliver therapeutic proteins to tumors, and this application model has been confirmed in clinical practice [40].

In addition to some of the above-mentioned microbial species found in abundance in the healthy samples, some microbes, with the aggravation of the disease, demonstrated a significantly increased abundance in the patients’ bodies compared to the healthy samples. For example, microbes such as *Dorealongicatena* and *Parabacteroides*, whose abundance was generally low or even non-existent in the healthy samples, was high in patients with adenoma and colorectal cancer. One hypothesis is that members of these genera may be harmful to the human body and cause imbalance in the human microbiota, causing the body to develop diseases such as adenoma and colorectal cancer. However, this is not the case. In the case of *Dorea longicatena*, which belongs to *Dorea*, this microbial species has been found to inhibit the progression of inflammatory diseases. When the body is experiencing inflammation, the microbial species increased correspondingly, producing butyrate and other anti-inflammatory molecules that are closely related to inflammatory colitis [41]. Therefore, it is important to identify key microbes closely related to disease and to determine their the abundance thresholds.

## 4. Conclusions

To improve upone existing random forest classification models, a mutual information method was introduced in this paper to screen features and remove features weakly correlated or unrelated to CRC, thus reducing the scale of the input data. The structure and information of all decision trees in the random forest were extracted to count and analyze the extent of the influence of various microbes on CRC to, in turn, determine the microbiota most closely related to CRC. Furthermore, the selected species in each decision for the decision trees and their corresponding abundance thresholds were used to explore the abundance threshold values of microbes associated with cancer.

To verify the feasibility and effectiveness of the above improved forest-based disease screening model, two metagenomics datasets of CRC samples were analyzed. After using GRAMMy to calculate the relative abundance and based on the extraction of internal information of the random forest model, the degree to which microbes impacted CRC in the sample data was further analyzed. The data presented in study may provide more streamlined criteria for clinical diagnosis and treatment; Which may also provide certain data to support clinical treatments such as fecal transplantation.

## Figures and Tables

**Table 1:** Schematic diagram of node information in decision tree.

Tree ID	Node Index	Father	Layer	Microbe ID	Split Value	Gini Index	Sample Number
0	0	0	0	324	0	0.4829	126
0	1	0	1	443	0.0006	0.3648	76
0	2	1	2	313	0.0001	0.4861	19
0	3	2	3	170	0.0001	0.4688	13
0	4	3	4	275	0	0.48	6
0	9	1	2	85	0	0.1818	54
0	11	9	3	428	0.0003	0.0907	51
0	13	11	4	429	0	0.2975	14
0	16	0	1	167	0	0.4543	53
0	17	16	2	90	0.0031	0.1327	8
0	20	16	2	440	0.0034	0.3607	45
0	21	20	3	273	0.0011	0.2706	39
0	22	21	4	319	0.0027	01349	35
0	25	21	4	458	0	0.2449	4
0	28	20	3	132	0.002	0.42	6
0	29	28	4	233	0.0002	0.2188	5

**Table 2:** Examples of the number of occurrences in various decision tree layers and the overall score of features.

Layer	0	1	2	3	4	Score	Microbial Species
Microbe ID							
334	213	262	270	239	181	232.437	*Porphyromonas asaccharolytica*
200	168	160	146	122	111	154.21	*Eubacterium hallii*
324	151	156	155	134	94	146.661	*Parvimonas* oral
220	177	127	129	117	80	145.268	*Fusobacterium* 7
350	144	147	157	131	132	144.319	*Prevotella melaninogenica*
443	117	149	170	151	136	136.618	*Streptococcus vestibularis*
343	119	129	161	138	122	129.228	*Prevotella copri*
332	130	138	118	118	89	125.879	*Peptostreptococcus stomatis*
226	131	128	125	115	68	123.025	*Fusobacterium nucleatum*
323	135	122	81	133	107	122.101	*Parvimonas micra*
233	117	104	123	103	111	112.955	*Gemella morbillorum*
213	82	130	131	142	125	109.201	*Flavonifractor plautii*
217	135	103	82	78	47	107.802	*Fusobacterium* 21
139	103	113	111	96	82	104.154	*Clostridium* SS2

**Table 3:** Information about the two sample groups.

Study Population	Healthy	Adenoma		Colorectal Cancer					Country of Residence
		Small (<1 cm)	Large (≥1 cm)	Early Stage			Late Stage		
				0	I	II	III	IV	
F (*N* = 156)	61	27	15	0	15	7	10	21	France
A *(N* = 156)	63	47		46					Austria

**Table 4:** Microbial species with high scores and abundance thresholds.

Microbe ID	Microbial Species	Score	Abundance Thresholds
334	*Porphyromonas asaccharolytica*	232.437	3.052 × 10^−5^
200	*Eubacterium hallii*	154.21	0.006662
324	*Parvimonas* sp.	146.661	1.391 × 10^−5^
220	*Fusobacterium* 7	145.268	0
350	*Prevotella melaninogenica*	144.319	0
443	*Streptococcus vestibularis*	136.618	0.0006701
343	*Prevotellacopri*	129.228	0.000179
332	*Peptostreptococcus stomatis*	125.879	9.154 × 10^−5^
226	*Fusobacterium nucleatum*	123.025	9.15 × 10^−5^
323	*Parvimonas micra*	122.101	7.63 × 10^−5^
233	*Gemella morbillorum*	112.955	5.19 × 10^−5^
213	*Flavonifractor plautii*	109.201	9.83 × 10^−5^
217	*Fusobacterium* 21	107.802	0
139	*Clostridium* SS2	104.154	0.000912
